# Features of Action Potentials from Identified Thalamic Nuclei in Anesthetized Patients

**DOI:** 10.3390/brainsci10121002

**Published:** 2020-12-17

**Authors:** Jesús Pastor, Lorena Vega-Zelaya

**Affiliations:** Clinical Neurophysiology and Instituto de Investigación Biomédica, Hospital Universitario de La Princesa, C/Diego de León 62, 28006 Madrid, Spain; lorenacarolina.vega@salud.madrid.org

**Keywords:** centromedian nucleus, deep brain stimulation, extracellular recordings, microelectrode recordings, sorting spikes, ventral caudal nucleus, ventrointermedial nucleus

## Abstract

Our objective was to describe the electrophysiological properties of the extracellular action potential (AP) picked up through microelectrode recordings (MERs). Five patients were operated under general anesthesia for centromedian deep brain stimulation (DBS). APs from the same cell were pooled to obtain a mean AP (mAP). The amplitudes and durations for all 2/3 phases were computed from the mAP, together with the maximum (*dV_max_*) and minimum (*dV_min_*) values of the first derivative, as well as the slopes of different phases during repolarization. The mAPs are denominated according to the phase polarity (P/N for positive/negative). We obtained a total of 1109 mAPs, most of the positive (98.47%) and triphasic (93.69%) with a small *P*/*N* deflection (*V_phase_*_1_) before depolarization. The percentage of the different types of mAPs was different for the nuclei addressed. The relationship between *dV_max_* and the depolarizing phase is specific. The descending phase of the first derivative identified different phases during the repolarizing period. We observed a high correlation between *V_phase_*_1_ and the amplitudes of either depolarization or repolarization phases. Human thalamic nuclei differ in their electrophysiological properties of APs, even under general anesthesia. Capacitive current, which is probably responsible for *V_phase_*_1_, is very common in thalamic APs. Moreover, subtle differences during repolarization are neuron-specific.

## 1. Introduction

The human thalamus is a complex structure composed of more than 50 different nuclear groups [1]. However, in general, there are no anatomical landmarks to identify a nucleus at the millimetric range. Nevertheless, the identification of thalamic nuclei is very important to obtain a good functional outcome in deep brain stimulation (DBS) to optimize battery life and decrease secondary effects.

Numerous studies have shown that electrophysiological properties of neurons depend on a specific set of ionic conductances [2,3,4], explaining the specific morphology of the action potential (AP) in terms of duration, rectification, or dynamics [5,6,7,8].

Microelectrode recordings (MERs) are useful tools used during DBS surgery to identify deep nuclei [9]. In addition to MER, other physiological tests can be performed to identify nuclei, such as cellular responses to voluntary or passive movements, tactile stimulus, or paresthesia induced by electrical stimulation [10,11]. However, most of these responses (except for the response to tactile stimuli) are nonspecific, and all of them need the conscious collaboration of the patient. Nevertheless, we have recently shown that raw traces are different for different thalamic nuclei in anesthetized patients [12].

Nonetheless, most of the information obtained during MER in clinical practice is restricted to the mean frequency of discharge and firing pattern (e.g., tonic, phasic, or more or less paused) [12,13,14,15,16], considering the AP as a bimodal variable (present or absent) that is represented as a vertical line without an inner structure in binary plots, under the assumption that the morphology of an AP does not carry any information [17]. However, we hypothesize that identified thalamic nuclei have different electrophysiological properties of extracellular APs recorded by MER during DBS. The aim of our study was to characterize the morphological properties of APs obtained from recordings picked up from identified thalamic nuclei. Identifying a specific pattern for every nucleus could help to identify neural structures during DBS surgery.

The preliminary results have been published in abstract form [18]. 

## 2. Methods

For this work, we followed the Schaltenbrand–Wahren (SW) atlas [19] nomenclature. What is called the parafascicular/centromedian Pf/CM in the literature is the centromedian (Ce) of ventroposterolateral and ventroposteromedial nuclei (VPL/VML) and are equivalent to ventrocaudal (V.c) [12].

### 2.1. Patients

We studied five patients undergoing DBS treatment for refractory generalized epilepsy in the Ce. The experimental procedure was approved by the medical ethics review board of the Hospital Universitario de La Princesa and was deemed “care as usual”. Under these circumstances, written informed consent was not required. Patients were initially assessed for study suitability using a presurgical evaluation in our center [20,21] and were excluded for resective surgery. See Table 1 for the clinical information.

### 2.2. Surgical Procedures

All of the patients were operated on while under general anesthesia using propofol (5.48 ± 0.28 mg/kg/h, (4.5, 6.2)) and remifentanil (0.12 ± 0.02 µg/kg/min, (0.1, 0.2)), maintaining a bi-spectral index (BIS) between 40 and 45. Neuromuscular blockade was accomplished with cisatracurium (0.5 mg/kg).

The thalamus was identified using a 1.5 T MRI (General Electric^®^, Fairfield, CT, USA), and the coordinates were located stereotactically with a neuronavigation (BrainLab^®^, Feldkirchen, Germany). The coordinates were calculated by fusing the MRI image and CT scan according to the SW atlas. For thalamic DBS electrode placement, a tentative initial target was selected in the Ce (x = 8, y = −10, z = 0). All of the coordinates refer to the mid-intercommissural anterior commissure–posterior commissure (AC–PC) line. Neuronal recordings (LeadPoint^®^, Minneapolis, MN, USA) were obtained beginning 10 mm above the target and progressing in steps of 0.5 mm, MERs (FHC^®^, Cumberland, ME, USA) were obtained until the inferior border of the thalamus was confirmed by the absence of neuronal activity. Impedance was always above 900 kΩ (1696 ± 80 kΩ, (900, 2900)).

**Figure A1:**
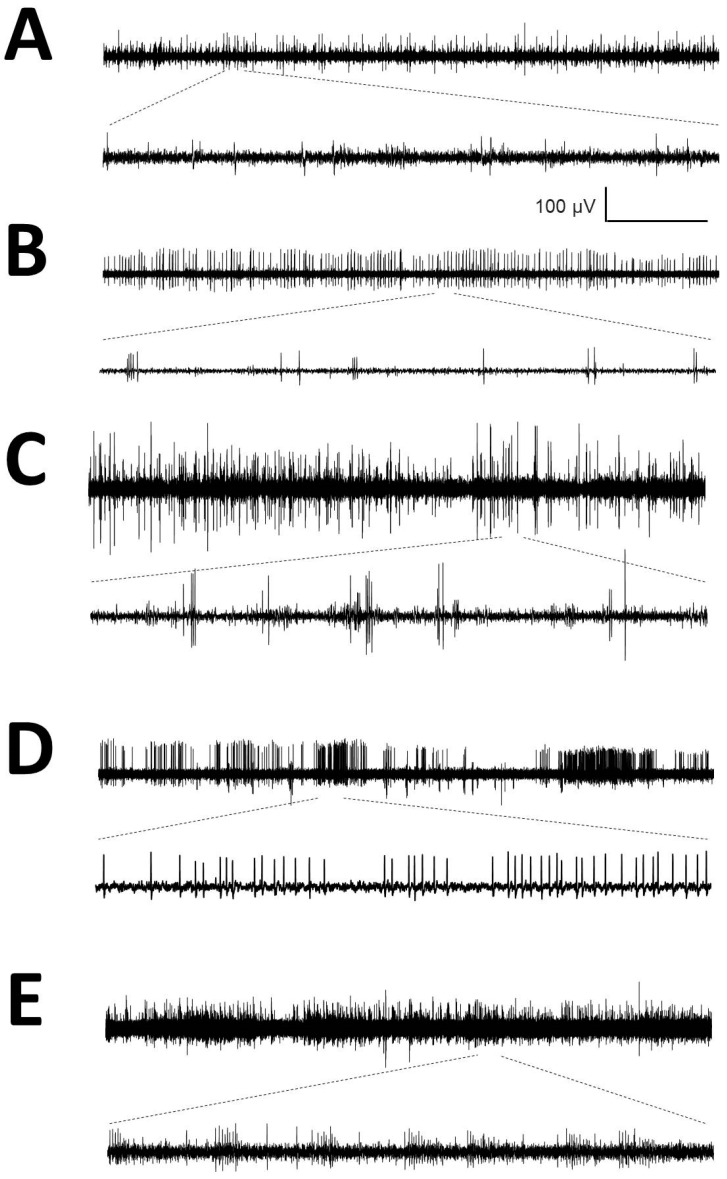
Raw traces from microelectrode recordings (MER) from different nuclei identified by reconstruction (**A**) Ce.pc; (**B**) Ce.mc; (**C**) V.im; (**D**) V.c and (**E**) DDNN. Horizontal calibration bar: 5 s for the upper traces and 167 ms for the lower traces.

MERs (see Figure A1, Appendix A) were obtained through four microelectrodes separated by 2 mm and placed (usually) at anterior, center, posterior, and lateral locations, except in patient 5, in whom posterior electrodes were replaced by medial ones. A microdrive was fixed to a stereotactic Leksell Coordinate Frame (Elekta^®^, Stockholm, Sweden). The bandwidth for spontaneous activity was 200 Hz–5 kHz, with a sample rate of 24 kHz. The notch filter was off. 

After the Ce was identified, a quadripolar DBS electrode was implanted, with a programmable stimulator placed in a pectoral or abdominal location. 

### 2.3. Reconstruction of the Trajectory

The reconstruction was described in detail elsewhere [22]. Briefly, we consider coordinate z = 0 the last recording inside the thalamus defined by the presence of an AP. Anteroposterior and lateral coordinates were obtained from the post-op MRI performed one month after surgery. Using this point and the stereotactic angles, we reconstructed the real trajectory of the electrode in a three-dimensional space in 1-mm intervals. Therefore, with the use of the SW map, we were able to identify in which nucleus each electrode was located throughout a trajectory.

### 2.4. Sorting Spikes and Analysis of Action Potentials

Data were exported as American Standard Code for Information Interchange ASCII files, and analyses were performed off-line. The recordings spanned 30–90 s (72−216 × 10^4^ points). Raw recordings were digitally filtered at 500 Hz–5 kHz using a 6th-order Butterworth. We used zero-phase forward and reverse digital infinite-impulse response (IIR) filtering [23].

The polarity of the potentials was defined as positive (*P*) upward and negative (*N*) downward, and was identified by order of appearance.

**Figure A2:**
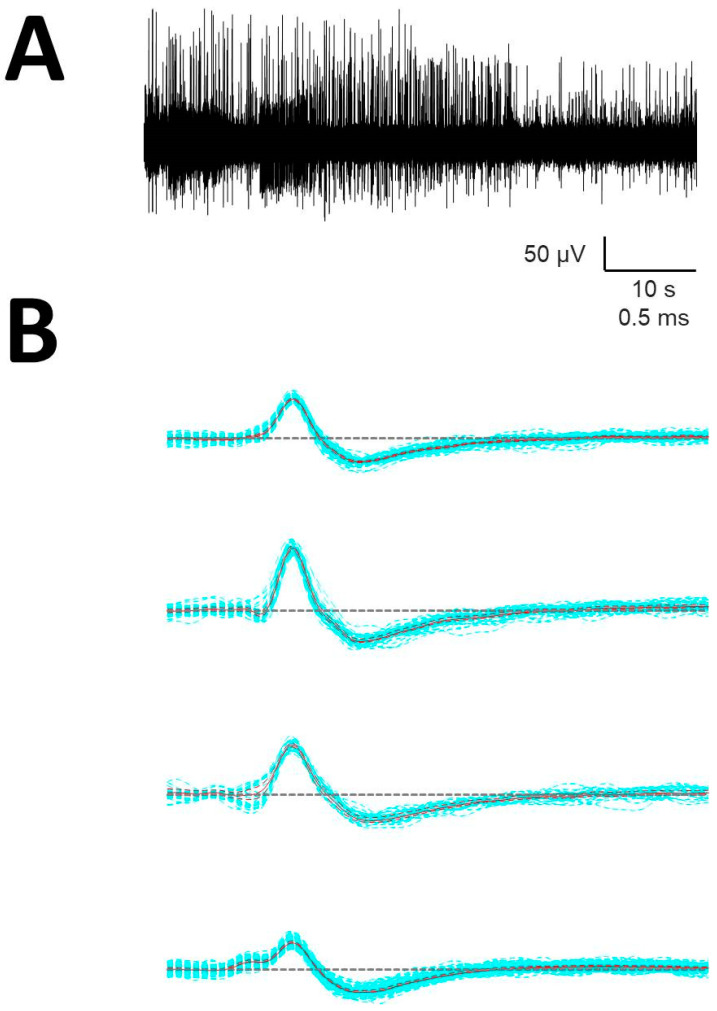
Sorting of spikes (**A**) Raw recording. (**B**) Units obtained from that trace. Blue: individual AP, continuous red = mean of all AP (mA), discontinuous red = upper/lower limits, dashed horizontal: basal level.

Sorting (see Figure A2, Appendix A) has been performed capturing features from the spike shapes and later used for clustering the waveforms. The more discriminative features, the better the ability to distinguish the different spike shapes [24] 

**Figure 1:**
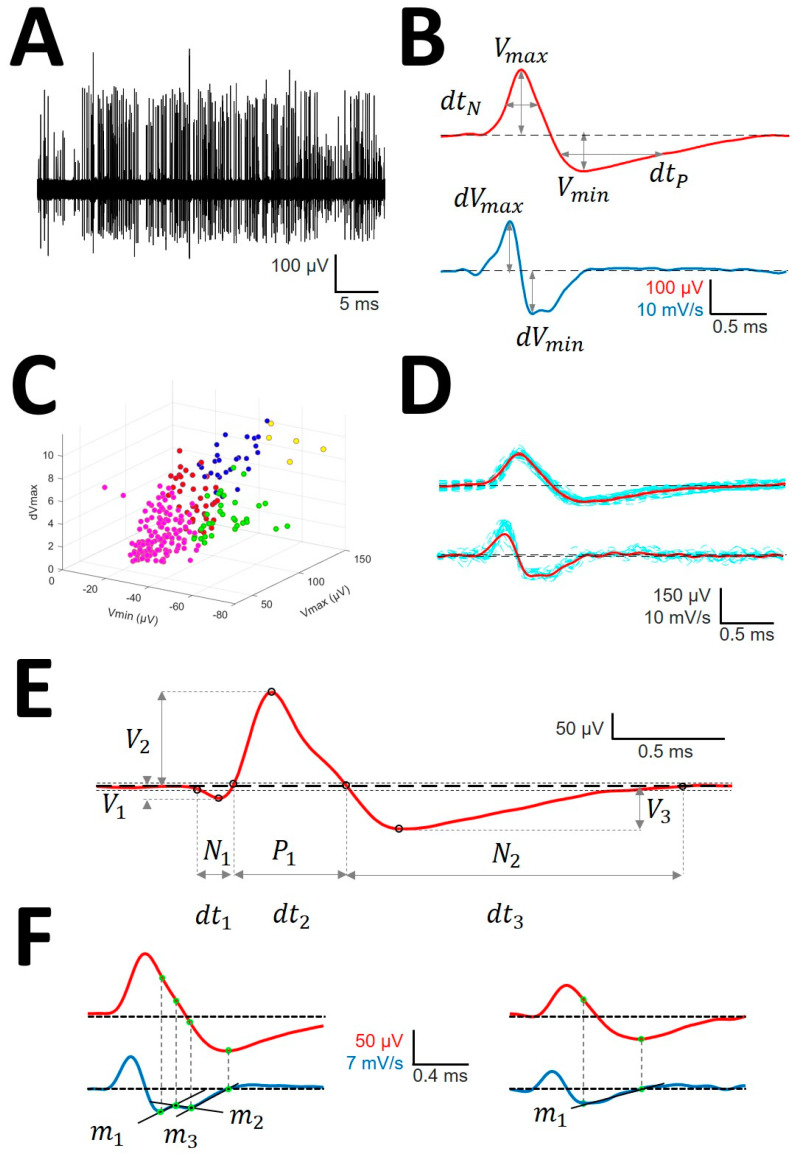
Sorting and analysis of action potential (AP) (**A**) Raw recording showing AP as vertical lines of different amplitude; (**B**) AP (upper row) and first derivative (lower row) showing the definitions of variables computed for sorting; (**C**) clustering in different units (identified by different colors) of the raw trace. Only three dimensions are shown, although clusterization used up to 6. (**D**) Mean AP (mAP) (upper row) and mean derivative (mDAP) (lower row) in red obtained from AP and dAP of similar morphology (blue lines). (**E**) Example of mAP showing the definition of variables analyzed. Tick dashed line represents basal voltage, and thin dotted lines represent upper and lower thresholds used to characterize the structure. Dots are the fiducial points used to define amplitudes and phases. (**F**) mAP (red, upper row) and mDAP (blue, lower row) showing different phases during repolarizing period. The left column shows a mAP with three different phases fitted to functions with different slopes (*m*_1_, *m*_2_, and *m*_3_) and the right one shows as mAP with only one phase (*m*_1_).

Algorithm for analysis:

1. Identification of APs. For every trace (Figure 1A), we computed a maximum (*V_+_*) and minimum (*V_−_*) voltage threshold (in µV), defined as $V_{\pm }=\bar{V}\pm 3.5\sigma_{V}$, where $\bar{V}$ is the mean and $\sigma_{V}$ is the standard deviation. APs must have two phases (depolarization and repolarization); therefore, we identified a tentative AP when a positive/negative (*P*/*N*) phase was followed by a negative/positive (*N*/*P*) phase in a period of 0.3–0.6 ms. APs were defined as positive $\left(\frac{P}{\left|N\right|}>1\right)$ or negative $\left(\frac{P}{\left|N\right|}<1\right)$ according to the highest component identified.
2. Clustering was performed by an agglomerative hierarchical method, with distance between groups computed by farthest procedure. APs sharing similar morphologies were ascribed to the same neuron. For every AP, we measured the maximum (*V_max_*) and minimum voltages (*V_min_*, in µV), durations of negative (*dt_N_*) and positive phases at half-amplitude (*dt_P_* in ms), and maximum (*dV_max_*) and minimum values of the first derivative (*dV_min_*, in mV/s). These measures can be considered as a 6-dimension vector for every *k*-AP, $AP_{k}=\left\{V_{max}^{k},V_{min}^{k},dt_{N}^{k},dt_{P}^{k},dV_{max}^{k},dV_{min}^{k}\right\}$ (Figure 1B). Then, we clustered the APs with similar properties using the standardized Euclidean distances (see below) (*d_E_*) (Figure 1C) [25].
3. Construction of the mean action potential (mAP). All of the APs from the same cluster were averaged to obtain a canonical waveform (Figure 1D, upper row), as were the derivatives to obtain the mean derivative (mDAP, 1D, lower row). A minimum of 10 APs were averaged. The first 300 µs (72 points) of baseline were used to compute the maximum (*V_AP+_*) and minimum (*V_AP−_*) voltage thresholds (in µV), defined as $V_{AP\pm }={\bar{V}}_{AP}\;\pm 2.5\sigma_{AP}$, where ${\bar{V}}_{AP}\;$is the mean and $\sigma_{AP}\;$the standard deviation. We used these thresholds to identify hallmark points in mAPs (Figure 1E). Every phase can be characterized by its polarity (P/N), duration (*dt_i_*), and amplitude (*V_i_*, *i* = 1, 2, 3).
4. Rectification of the repolarizing phase. We analyzed the number of phases and their slopes. The local maxima and minima of the mDAP between the lowest value and the zero crossing were taken to define uniform dynamics in the mAP (see Figure 1F). We used two consecutive points, *i,j*, in mDAP ($\left(x_{i},y_{i}\right),\;\left(x_{j},y_{j}\right)$) to find the slope (*m*) following the formula:
  (1) $$m=\frac{y_{j}-y_{i}}{x_{j}-x_{i}}$$

### 2.5. Evaluation of Global Similarity

We computed the distance (e.g., dissimilarity) between every pair of nuclei. Therefore, the smaller the distance, the greater the similarity. We used the standardized Euclidean distance (*d_E_*) [26]. First, we computed the covariance matrix (*S*), with variances ($s_{i}^{2}$) at the main diagonal. For every pair of elements (*i,j*) we computed:

(2) $$d_{E,ij}={\left[\left(AP_{i}-AP_{j}\right){}^{\prime }S^{-1}\left(AP_{i}-AP_{j}\right)\right]}^{1/2}$$

where $\left(AP_{i}-AP_{j}\right){}^{\prime }$ is the transpose vector of $\left(AP_{i}-AP_{j}\right)$. 

The same metrics were used with other vectors different from *AP*.

All analyses were performed using homemade MATLAB^®^R2019 (MathWorks, Natick, MA, USA) scripts.

### 2.6. Classification of mAP According to Morphology

To compare in detail the structure of mAP, we analyzed different properties (e.g., *V_max_*, *V_min_*, *dt_N_*, etc.) for the main four types of mAP by means of ANOVA on ranks and post-hoc analysis between pairs of variables was performed. We considered the largest first phase to be depolarization; while the second largest phase corresponds to repolarization. This definition is reinforced by the diverse duration of the two phases, which are always shorter for depolarizations than for repolarizations. Therefore, in case of positive cells (see below), depolarization corresponds to N1 and repolarization to P2. However, in this kind of mAP, the polarity is opposite to that of the others; thus, we inverted every AP (multiplying by −1) to allow a comparison with the rest of the types.

### 2.7. Statistics

Kurtosis (K) was computed for every group, and only values between 2 and 8 were acceptable for the homogeneous group [27]. Extreme outliers were removed. Statistical analysis was applied only to these groups. 

Statistical comparisons between groups were performed using the Student’s *t*-test or Kruskal–Wallis One Way Analysis of Variance on Ranks if normality failed. In the last case, Dunn’s method was used for all pairwise post hoc comparisons. Normality was evaluated using the Kolmogorov–Smirnov test. The Chi-square test ($\chi^{2}$) was used to assess differences between groups. To avoid values lower than unity (which decrease the value of $\chi^{2}$ instead of increasing the difference), comparisons between mAPs were performed on values normalized to the most frequent mAP (P1P2N1, see below). The independence of variables (e.g., peak-to-peak action potential amplitude and amplitudes of depolarizing and repolarizing phases) was assessed by computing the rank (*rnk*) for the matrix containing observations (*n*) in rows and variables (*p*) in columns. Therefore, if *rnk < p* (considering that *n > p* always), then there would be some dependent variable that could be removed. However, when *rnk = p*, all the variables are independent and must be included in the analysis. 

SigmaStat^®^ 3.5 software (Aspire Software, Richmond, CA, USA) and MATLAB (The MathWorks, Natick, MA, USA) were used for statistical analyses.

Pearson’s correlation coefficient was used to study the linear dependence between variables. Linear regression significance was evaluated by means of a contrast hypothesis against the null hypothesis *ρ* = 0 using the formula

(3) $$t=\frac{r\sqrt{n-2}}{\sqrt{1-r^{2}}}$$

This describes a *t*-Student distribution with *n* − 2 freedom degrees. The slope of a linear function (*m*) can be statistically compared with a definite value *A*, making use of the fact that the statistics [28]

(4) $$t=\frac{m-A}{s_{y.x}/s_{x}}\sqrt{n-2}$$

follow the *t*-Student distribution with *n* − 2 degrees of freedom. We can define $s_{y.x}=\sqrt{\frac{\sum_{i=1}^{N}{\left(y_{i}-y_{e,i}\right)}^{2}}{n-2}}$ as the standard deviation of estimate and $s_{x}=\sqrt{\frac{\sum_{i=1}^{N}{\left(x_{i}-\bar{x}\right)}^{2}}{n-2}}$.

The significance level was set at *p* = 0.05 and the results are shown as the mean ± SEM.

## 3. Results

### 3.1. Reconstruction of Trajectories

For every position, we identified the nucleus from which the trace originated (see [22]). We focused on the following nuclei: Ce, V.c, ventrointermedial (V.im), ventral oralis (V.o), and dorsal, collectively addressed as (DDNN for dorsal nuclei) (e.g., dorsalis intermedius or ventrointermedius). In total, the trajectories assigned to different nuclei and computed from all the electrodes measured 171 mm in V.c, 99 mm in Ce, 83 mm in V.im, 15 mm in V.o, and 72 mm in DDNN. 

All of the properties analyzed were obtained from mAPs and mDAPs. Ce is divided into magno (Ce.mc) and parvocellular (Ce.pc).

### 3.2. Types of mAP According to Structure

We obtained a total of 139 mAPs for Ce.pc, 112 for Ce.mc, 221 for V.im, 528 for V.c, and 109 for DDNN, for a total of 1109. Bearing in mind that every mAP is composed of 28.8 ± 11.6 APs, the total number of individual APs analyzed was greater than 32,000.

**Figure 2:**
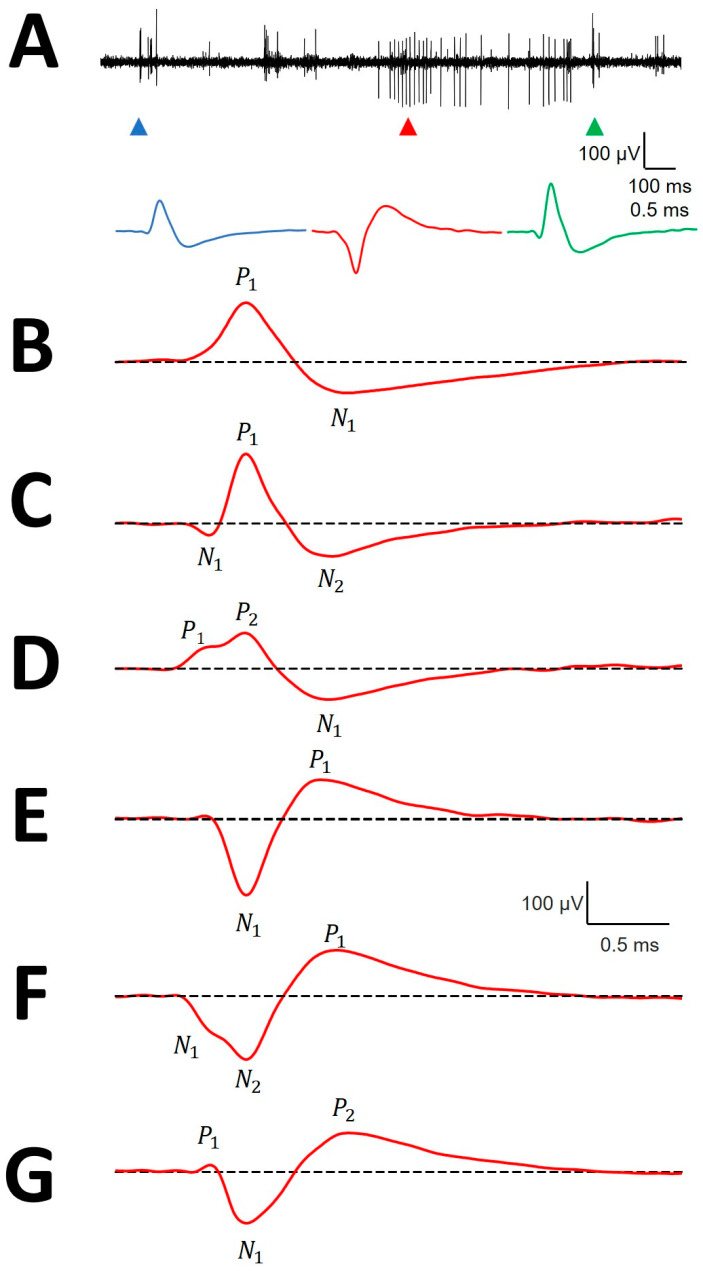
Canonical forms of mAPs. (**A**) Simultaneity of positive and negative extracellular action potentials. Upper row: raw trace containing upward and downward directed potentials; bottom row: AP corresponding to the upper arrowheads. Each colored P corresponds to the same color arrowhead. (**B**) P1N1; (**C**) N1P1N2; (**D**) P1P2N1; (**E**) N1P1; (**F**) N2N2P1 and (**G**) P1N1P2. The dashed line indicates the basal voltage. Positive (*P*) and negative (*N*) phases are indicated.

We analyzed the structure of mAPs, defined as the arrangement of the parts composing the entire waveform. Most of the mAPs were positive, 1092/1109 (98.47%), while negative ones were scarce. Negative mAPs were recorded at the same places as positive ones (Figure 2A). Only 70/1109 (6.31%) mAPs showed 2 phases. The most frequent had either a small positive or negative deflection before the main component (Figure 2C,D,F,G), which yielded a three-phase structure for 93.69% of mAPs. In 806/1109 (72.68%) cases, a P1P2N1 structure was observed, followed by a N1P1N2 structure in 216/1109 (19.48%) and a P1N1P2 structure in the remaining 17/1109 (1.53%). Some of the mAPs shown in Figure 2 (specifically panels E and F) are anecdotal and are shown to complete the picture but were not numerically analyzed. The numerical properties of P1N1 are shown at Table A1, the properties of P1P2N1 at Table A2, the properties of N1P1N2 at Table A3, and the properties of P1N1P2 at Table A4, all of them at Appendix B.

No differences in the types of mAPs between nuclei were observed. Thus, instead of a nucleus-specific analysis, we analyzed the kind of mAPs, grouping all the mAPs that shared the same structure, irrespective of the nucleus from which they were selected.

**Figure 3:**
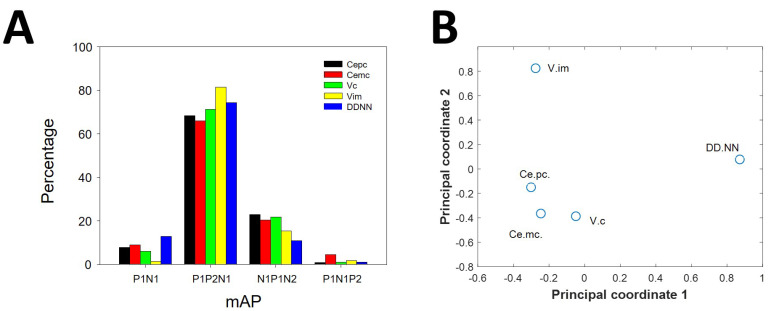
Distribution of mAP among nuclei. (**A**) Bar graph showing the presence of different kind of mAP for nuclei (black = Ce.pc; red = Ce.mc; green = V.c; yellow = V.im and blue = DDNN). (**B**) Multidimensional scaling analysis showing similarities between nuclei by types of mAP. Therefore, the composition of mAP, e.g., the different proportion of types of mAP, was different for every nucleus, except for both parts of Ce, which exhibited a similar composition.

The percentages of different types of mAPs were different for the nuclei considered (Figure 3A). The distribution of types of mAPs was different (3 degrees of freedom) when comparing these pairs of nuclei: Ce.pc/V.im ($\chi^{2}$ = 11.62, *p* < 0.01), Ce.pc/DDNN ($\chi^{2}$ = 9.87, *p* < 0.05), Ce.mc/V.im ($\chi^{2}$ = 12.75, *p* < 0.01), V.c/DDNN ($\chi^{2}$ = 13.1, *p* < 0.01), and V.im/DDNN ($\chi^{2}$ = 95.0, *p* < 0.001). In contrast, only the distributions of Ce.pc and Ce.mc were similar ($\chi^{2}$ = 3.46, n.s). We used multiscale analysis and principal coordinates analysis [25] to evaluate the similarity between nuclei. We plotted these relationships as a bidimensional graph (Figure 3B), using the two principal coordinates. As can be observed, Ce.mc and Ce.pc are closely related; therefore, they are composed of similar percentages of different types of APs. However, both are closely similar to V.c and in a lesser degree to V.im. The most different composition was observed for DDNN. 

### 3.3. Canonical Description of mAP

We considered the largest first phase to be depolarization; while the second largest phase corresponds to repolarization. 

The assessment of the global similarities of the properties of mAPs between normalized groups using the $\chi^{2}$ test (7 degrees of freedom) showed highly significant differences for all the analyzed pairs, i.e., P1P2N1/P1N1 ($\chi^{2}$ = 182.56, *p* < 0.001), P1P2N1/N1P1N2 ($\chi^{2}$ = 20.98, *p* < 0.01), P1P2N1/P1N1P2 ($\chi^{2}$ = 27.70, *p* < 0.01), P1N1/N1P1N2 ($\chi^{2}$ = 105.40, *p* < 0.001), P1N1/P1N1P2 ($\chi^{2}$ = 154.23, *p* < 0.001), and N1P1N2/P1N1P2 ($\chi^{2}$ = 21.04, *p* < 0.01).

**Figure 4:**
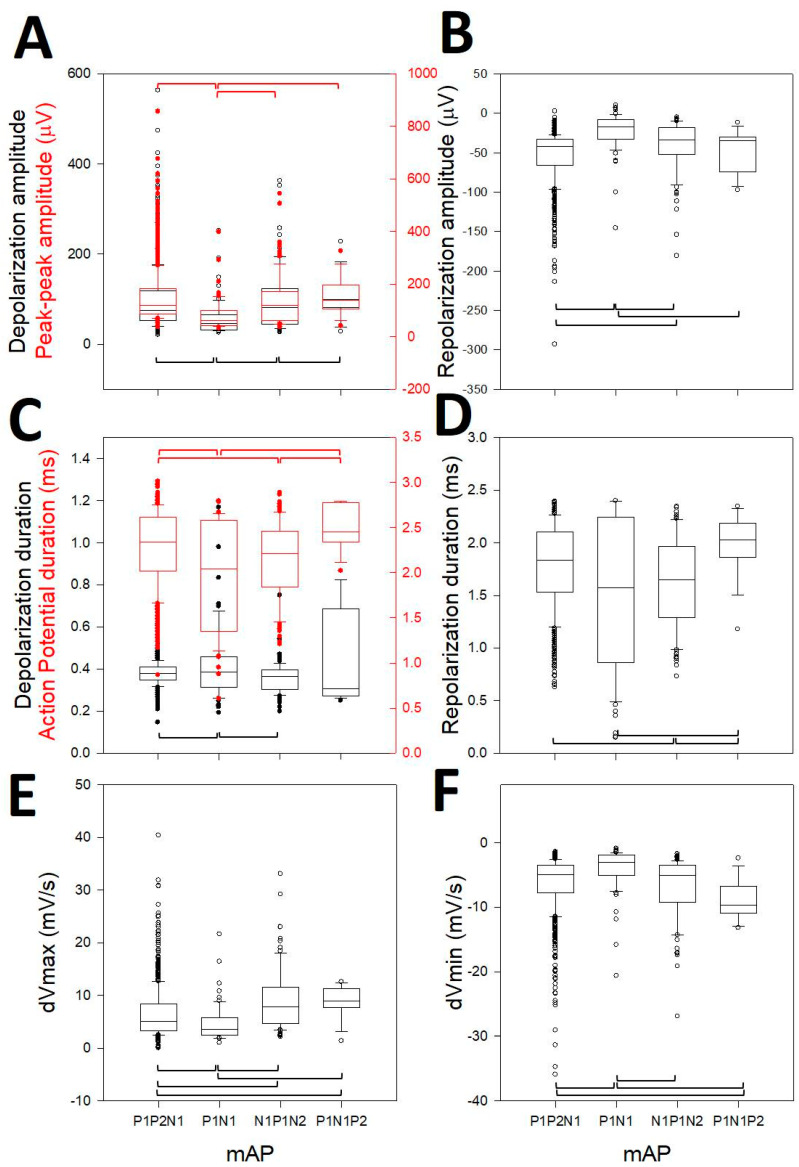
Box-plot graphs showing different properties of mAPs, indicated at *x*-axis. (**A**) Depolarization (black) and peak-to-peak (red) amplitudes; (**B**) amplitude repolarization; (**C**) depolarization (black) and action potential (red) duration; (**D**) repolarization duration; (**E**) dVmax and (**F**) dVmin. Horizontal brackets indicate the difference (Kruskal–Wallis One Way Analysis of Variance on Ranks, Dunn’s test post-hoc) with post hoc test between pairs of variables. Red brackets refer to whole action potential variables (peak-to-peak and total duration).

We observed that all of the properties analyzed were highly different according to ANOVA on ranks (*p* < 0.001), although they were not different for all the pairwise post-hoc comparisons, but were for the majority of them (Figure 4). In this sense, the depolarization amplitude was different for 3/6 pairs (Figure 4A, N1N2P1 vs. N1P1; N1P1 vs. P1N1P2; P1N1P2 vs. N1P1N2, black boxes), the peak-to-peak amplitude was different for 3/6 pairs (Figure 4A, N1N2P1 vs. N1P1; N1P1 vs. P1N1P2; N1P1 vs. N1P1N2, red boxes), the repolarization amplitude was different for 4/6 (Figure 4B, N1N2P1 vs. N1P1; N1N2P1 vs. P1N1P2; N1P1 vs. P1N1P2; N1P1 vs. N1P1N2), the depolarization duration was different for 2/6 (Figure 4C, N1N2P1 vs. N1P1; N1P1 vs. P1N1P2, black boxes), the AP duration was different for 4/6 (Figure 4C, N1N2P1 vs. N1P1; N1N2P1 vs. P1N1P2; N1P1 vs. N1P1N2; P1N1P2 vs. N1P1N2, red boxes), the repolarization duration was different for 3/6 (Figure 4D, N1N2P1 vs. P1N1P2; N1P1 vs. N1P1N2; P1N1P2 vs. N1P1N2), the *dV_max_* was different for 5/6 (Figure 4E, N1N2P1 vs. N1P1; N1N2P1 vs. P1N1P2; N1N2P1 vs. N1P1N2; N1P1 vs. N1P1N2; P1N1P2 vs. N1P1N2), and the *dV_min_* for was different 4/6 pairs (Figure 4F, N1N2P1 vs. N1P1; N1N2P1 vs. N1P1N2; N1P1 vs. P1N1P2; N1P1 vs. N1P1N2). The properties in this figure consider the opposite polarity for P1N1P2. Therefore, obviously, the true difference between P1N1P2 and the rest was even higher, considering that the depolarization and repolarization phases in these kinds of mAPs are inverted [29]. 

The similarity was also different between pairs of mAPs. We observed that cells with P1P2N1 are closely related to those with N1P1N2, but are different from those with P1N1.

### 3.4. Properties of the First Derivative

**Figure 5:**
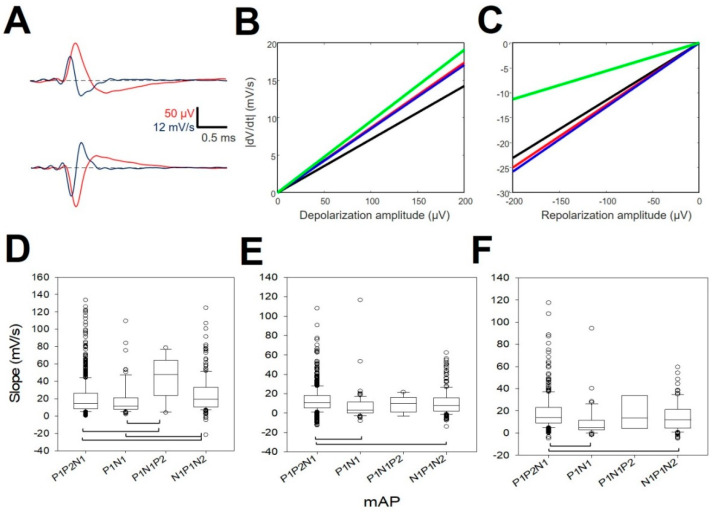
Properties of the first derivative. Relationships between voltage amplitudes and derivatives. (**A**) Examples of negative APs (upper row) and positive (lower row) APs (red) and their derivatives (blue). The maximum value of the |dV/dt| correspond to depolarization in both cases. (**B**) Linear regression between amplitude and dV/dt  for repolarization and (**C**) depolarization. P1P2N1 = black; P1N1 = red; N1P1N2 = blue and P1N1P2 = green. Box-plots showing the values of slopes for the three types of negative mAPs. (**D**) First phase slope (*m*_1_), (**E**) second phase slope (*m*_2_), and (**F**) third phase slope (*m*_3_). The horizontal brackets indicate the significant difference with post hoc test between pairs of variables (Kruskal–Wallis One Way Analysis of Variance on Ranks, Dunn’s test post-hoc).

We plotted the *dV_max_* versus maximum depolarizing amplitude and *dV_min_* versus minimum value of the repolarizing amplitude. The polarities are opposite for P1N1P2 mAPs (Figure 5A). We computed the linear regression between both variables using the least-square minimum method. Using Equation (4), we checked that all correlation coefficients were statistically significant with respect to the null hypothesis H_0_: *ρ*_0_ = 0. Only for the P1N1P2 repolarizing phase, where *r* = 0.5167, was the significance *p* < 0.05; for the other cases, *p* < 0.001 (one-tailed Student’s *t*-test). From Figure 5, we can observe that both the depolarizing (Figure 5B) and repolarizing slopes (Figure 5C) for P1N1 and N1P1N2 (red and blue lines, respectively) were similar. We computed the mean values for both and used equation 4 to assess the similarity with the slopes of the other mAPs. Both the depolarization and repolarization phases for P1P2N1 and P1N1P2 mAPs were different from (P1N1+N1P1N2)/2 (*p* < 0.001 for two-tailed Student’s *t*-test). 

Therefore, the relationship between *dV_max_* and the depolarizing phase is not an unspecific process, but on the contrary is dependent on the type of mAP. Only N1P2 and P1N1P2 shared similar functions with practically similar slopes.

We also analyzed the different phases during repolarization in mDAPs. Most of the mAPs (98.56 ± 0.59%) showed at least two phases, and almost half of them (47.69 ± 3.36%) showed three phases. Four phases were found anecdotic (5.66 ± 0.55%), and one phase was scarce (<1%). We did not find differences in the constitution of the number of phases for types of mDAPs. As can be observed from Figure 5D, the slope of the first phase was different for all the pairs, except for P1P2N1/P1N1 and P1N1P2/N1P1N2. However, phases 2 and 3 were more similar because we only observed differences between P1P2N1/P1N1 and P1P2N1/N1P1N2. Values for slopes are shown at Appendix B, Table A5.

Therefore, it can be concluded that the kinetics of the repolarization phase are different at least for the first slope of the repolarizing phase. However, the rest of the repolarization was more conservative, except for mAP P1P2N1.

### 3.5. Analysis of the First Phase

**Figure 6:**
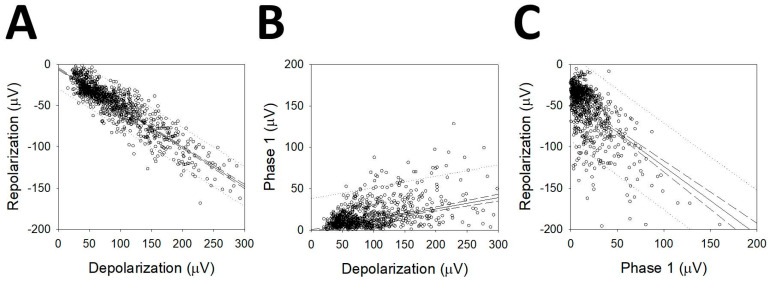
Linear regression for the maximum amplitude of the different phases of mAPs. (**A**) Depolarization vs. repolarization; (**B**) depolarization vs. phase 1 and (**C**) phase 1 vs. repolarization. Solid lines represent linear regressions; dashed lines represent 95% confidence band and dotted lines 95% prediction band.

We observed that most of the mAPs analyzed showed a first phase that was either positive or negative and smaller than the depolarizing phase. We correlated the maximum amplitudes of depolarization (*V_depol_*) and repolarization (*V_repol_*) (Figure 6A), and both of these phases with the first smaller one (*V_phase_*_1_). In the case of P1N1P2 cells, the amplitudes were multiplied by −1 so that their polarities were similar to those of the rest of the mAPs. The linear function adjusted was $V_{repol}\left(V\right)=-0.475V_{depol}-5.540$, *r* = 0.925 (*p* < 0.001, Student’s *t*-test). The linear regression between phase 1 and depolarization (Figure 6B) was $V_{phase1}\left(V\right)=0.136V_{depol}-1.678$, *r* = 0.390 (*p* < 0.001, Student’s *t*-test), and finally, the comparison between phase1 and repolarization (Figure 6C) gave $V_{repol}\left(V\right)=-0.829V_{phase1}-40.296$; *r* = 0.563 (*p* < 0.001, Student’s *t*-test). These results show that *V_phase_*_1_ is highly correlated with the other two phases forming the AP and, therefore, probably pertain at the same process.

Although the correlation was higher for *V_repol_/V_depol_*, it was also highly significant for *V_phase_*_1_, which means that there is a correlation between the first phase and the repolarization/depolarization phases.

## 4. Discussion

To the best of our knowledge, this is the first work showing that human thalamic nuclei differ in their electrophysiological AP properties. This does not mean that every thalamic nuclei have a specific type of mAP, but that a limited set of mAP, diverse in electrophysiological properties, is mixed at different percentage at each nucleus. This is an important clinical finding because it could permit the development of a way to specifically identify certain thalamic nuclei without anatomical references and does not need the conscious collaboration of the patient. Therefore, we could perform this type of surgery under general anesthesia, considering the patient’s preferences and well-being, allowing the reduction of stress and discomfort and avoiding awake surgery [30,31]. However, demonstration of this possibility was clearly out of the scope of this work and we merely mention this exciting option. Moreover, this could be also relevant for neuroscience, because is, not only the first work showing at this degree of complexity differences in AP structure extracellularly recorded, but this is done in humans. Therefore, morphology of AP can be useful to extract relevant information, besides the discharge pattern.

Different methods for spike sorting have been described [32,33,34,35,36]. However, there is not a recognized-best-method. In this sense, clustering is a well-known, robust, and straightforward approach to grouping sets of data [37,38]

AP width has been reported for the pedunculopontine nucleus in humans, and a bimodal distribution has been observed, with a longer AP attributed to cholinergic neurons and a shorter AP attributed to glutamatergic transmission [39,40]. However, no other properties have been analyzed (number of phases, features of phases, derivatives, etc.). In our work, we obtained APs that were either positive or negative, mostly with three phases. Multidimensional scaling analyses showed that subnuclei from Ce are closer (i.e., the AP features are more similar) than for the rest of the nuclei and also closer to V.c and were the most different from DDNN, which is expected because we pooled mAPs from different dorsal nuclei.

We showed in 1109 mAPs (more than 32,000 individual APs) that the specific properties of amplitude, duration, and change rates for the depolarization phase and, even more specifically, for the repolarization phase are highly different. In animal recordings, the temporal structure of extracellular APs contains information about the intracellular spike [29]; therefore, we can hypothesize that the ionic conductances are different for each type of mAP. It has been postulated that in the cortex, pyramidal neurons, and interneurons can be differentiated by the wider spike of the first [29,41,42]. We obtained a bimodal distribution amplitude and the duration of the positive phase of APs, which are longer and shorter for N1P1 than for N1N2P1 or P1N1P2, although this cleavage is not as clear for repolarizing or derivative properties. 

It has been shown that the amplitude of extracellular spikes decreases monotonically with the distance from the soma [17,29]. This fact can explain the variation in amplitude because we can record neurons from a sphere of tissue approximately 5.23 × 10^5^ µm^3^ in volume. However, not only the distance to the microelectrode can be argued to modify the amplitude, but the net local field potential can also affect the amplitude of an AP [29,42]. In fact, the total extracellular current injected to the volume recorded (e.g., by sources/sinks from surrounding tissue) can affect the amount of extracellular voltage generated by an extracellular current caused by an AP. Nevertheless, in spite of these sources of variation, we have obtained highly significant differences in amplitude between types of mAP. 

A very interesting observation is that repolarization can be composed of different phases, and this difference, instead of being randomly distributed, is specific for different types of mAPs. Simultaneous intra-extracellular recordings have shown that depolarization from intracellular recordings is longer than from extracellular recordings; in fact, the first segment of the ascending phase after the minimum voltage value (i.e., the maximum amplitude of what we have termed depolarization) in an extracellular AP occurs during the depolarizing phase of an intracellular AP [29,42]. In fact, the maximum value of depolarization for intracellularly recorded APs coincides with a notch in the ascending phase of extracellular APs. This effect is observed mainly when simultaneous extra- and intracellular recordings are near the cellular soma. A possible explanation for this result is the variation in the relative position of microelectrodes and the cellular soma. However, in that case, we would expect that random variation of the relative distance would be the only source of difference that cannot produce the significant differences observed for different mAPs. Therefore, although we obviously cannot exclude the differences of relative positions of microelectrode and soma as source of variations, we postulate that differences in ionic conductances in cells can also contribute to these changes in dynamics during the repolarization phase.

We observed the presence of a phase before depolarization in 93.69% of neurons. This first phase likely corresponds to the capacitive current. In fact, both numerical and experimental data have shown the presence of capacitive current prior to the large depolarizing phase [17,42,43,44]. We observed a highly significant correlation between the first phase amplitude and the two other phases of mAPs; therefore, this small waveform is causally related to the action potential. It could be speculated that postsynaptic potentials may be responsible for this phase. However, if this were the case, then a greater variation would be expected, as would an absence of correlation with the other phases. However, the capacitive current is elicited by current spreading to dendrites from the soma and is always of the opposite polarity than the depolarization [17,42,43]. Surprisingly, we observed both opposite and similar polarities, which are difficult to collate with the capacitive current.

Another result is difficult to explain: the simultaneous presence of positive and negative cells. We must assume that the faster (in dynamics) and shorter phases of APs correspond to depolarization and that the following, usually slower and longer phases correspond to repolarization [29,42,44]. Numerous lines of empirical evidence show that depolarization is caused by the inward transmembrane current, while the outward current is responsible for repolarization (see 7 for a review), and numerical simulations have shown that the shape of the AP is proportional to the total transmembrane current of perisomatic compartments [17,42,43]. Therefore, despite changes in the dendritic morphology, depolarization must always be driven by the inward Na^+^ current; therefore, they must be positive for our arrangement. However, we recorded mAPs with opposite polarities, although in an extremely low percentage (1.53%). Considering that the amplifier reference electrode is located in the same place, the only explanation for a negative depolarization phase is a transmembrane outward current. In animal cortical recordings, high amplitude positive action potential different from conventional negative spikes has been described [45]. Features of both kinds of spikes are clearly different, such as in amplitude as duration. However, that is not our case, were properties of positive and negative mAP (absolute magnitudes for amplitudes and phases durations) are only slightly different, but in the same order of magnitude. Besides, there is no reason to consider that cortex and deep brain nuclei neurons share similarities in ionic conductances or morphology.

## 5. Conclusions

We have shown for first time that human thalamic nuclei differ in their electrophysiological properties of APs, even under general anesthesia. This property can be used to positively identify thalamic nuclei without the necessity of conscious help by the patient or morphological references. Additionally, capacitive current, which is probably responsible for *V_phase_*_1_, is very common in thalamic APs. Moreover, subtle differences during repolarization are specific for every type of neuron.

## Figures and Tables

**Table 1:** Clinical features of the patients.

Patient	Gender	Age (Years)	History (Years)	Etiology	v-EEG	MRi	VNS
#1	F	37	31	Genetic ^1^	GE	Normal	Yes
#2	F	18	12	LGS	GE	Dysplasia LF	No
#3	M	30	23	Structural	GE/EE	Dysplasia biFT	Yes
#4	M	34	27	Genetic ^2^	EG/EE	Normal	Yes
#5	M	27	27	LGS	GE	Normal	No

F: female. M: male. EE: epileptic encephalopathy. GE: generalized epilepsy. LF: left frontal. biFT: bilateral frontotemporal. LGS: Lennox–Gastaut syndrome. MRi: magnetic resonance imaging; v-EEG: video-electroencephalography. VNS: vagus nerve stimulation. ^1^ 20 ring-chromosome syndrome. ^2^ Tuberous sclerosis.

## Appendix B

Numerical properties of mAPs at different nuclei. Abbreviations are the same for all tables; therefore, they are shown only at end of Table A4.

**Table A1:** Properties of mAP for P1N1. Values as $\bar{x}\pm SEM$, except of *N*, which indicates the number of mAP picked up from every nucleus.

Properties	Ce.pc	Ce.mc	V.c	V.im	DD.NN
N	11	10	32	3	14
N1 (µV)	47.6 ± 7.7	40.0 ± 4.1	51.0 ± 4.3	48.9 ± 12.3	86.2 ± 18.0
P1 (µV)	−15.5 ± 5.7	−18.4 ± 3.2	−16.1 ± 3.1	−17.2 ± 9.7	−43.7 ± 10.2
durN1 (ms)	0.38 ± 0.03	0.41 ± 0.05	0.49 ± 0.04	0.34 ± 0.04	0.37 ± 0.03
durP1 (ms)	1.59 ± 0.04	1.41 ± 0.23	1.49 ± 0.13	1.53 ± 0.43	1.64 ± 0.19
Peak-peak (µV)	63.7 ± 12.3	58.4 ± 6.8	69.0 ± 6.6	67.5 ± 20.1	129.8 ± 27.8
durPA (ms)	1.97 ± 0.24	1.82 ± 0.19	2.00 ± 0.11	1.87 ± 0.40	2.13 ± 0.17
dVmax (mV/s)	4.3 ± 0.8	3.5 ± 0.4	3.9 ± 0.4	4.1 ± 1.2	7.2 ± 1.6
dVmin (mV/s)	−3.5 ± 0.74	−3.1 ± 0.4	−3.4 ± 0.4	−2.8 ± 0.9	−6.5 ± 15

**Table A2:** Properties of mAP for P1P2N1. Values as $\bar{x}\pm SEM$, except of *N*, which indicates the number of mAP picked up from every nucleus.

Properties	Ce.pc	Ce.mc	V.c	V.im	DD.NN
N	95	74	376	180	81
N1 (µV)	19.5 ± 2.1	13.9 ± 1.6	15.2 ± 0.7	24.7 ± 1.9	19.4 ± 1.4
N2 (µV)	89.6 ± 6.4	95.5 ± 7.5	89.8 ± 3.0	111.4 ± 5.8	91.3 ± 5.3
P1 (µV)	−50.1 ± 3.3	−49.6 ± 1.5	−51.6 ± 1.6	−62.9 ± 2.9	−54.8 ± 2.9
durN1 (ms)	0.11 ± 0.01	0.11 ± 0.01	0.12 ± 0.00	0.12 ± 0.01	0.12 ± 0.01
durN2 (ms)	0.37 ± 0.01	0.38 ± 0.01	0.38 ± 0.00	0.40 ± 0.00	0.39 ± 0.01
durP1 (ms)	1.68 ± 0.05	1.80 ± 0.02	1.80 ± 0.02	1.85 ± 0.02	1.71 ± 0.05
Peak-peak (µV)	139.4 ± 9.6	139.3 ± 4.4	144.5 ± 4.6	174.2 ± 8.6	146.1 ± 8.0
durPA (ms)	2.15 ± 0.05	2.28 ± 0.02	2.29 ± 0.02	2.37 ± 0.03	2.23 ± 0.05
dVmax (mV/s)	6.0 ± 0.5	6.5 ± 0.3	6.6 ± 0.03	7.4 ± 0.4	6.2 ± 0.4
dVmin (mV/s)	−6.6 ± 0.5	−6.0 ± 0.2	−6.2 ± 0.2	−6.9 ± 0.4	−6.0 ± 0.4

**Table A3:** Properties of mAP for P1N1P2. Values as $\bar{x}\pm SEM$, except of *N*, which indicates the number of mAP picked up from every nucleus.

Properties	Ce.pc	Ce.mc	V.c	V.im	DD.NN
N	32	23	115	34	12
P1 (µV)	−12.5 ± 2.0	−15.1 ± 2.3	−15.8 ± 0.9	−17.8 ± 2.2	−17.1 ± 2.4
N1 (µV)	60.3 ± 6.1	80.5 ± 14.8	79.1 ± 4.6	119.2 ± 12.9	122.8 ± 19.8
P2 (µV)	−23.8 ± 2.7	−34.9 ± 7.0	−34.5 ± 2.1	−51.5 ± 5.6	−56.9 ± 10.8
durP1 (ms)	0.13 ± 0.01	0.17 ± 0.02	0.16 ± 0.01	0.15 ± 0.01	0.17 ± 0.03
durN1 (ms)	0.34 ± 0.01	0.32 ± 0.01	0.37 ± 0.01	0.37 ± 0.01	0.40 ± 0.04
durP2 (ms)	1.51 ± 0.08	1.70 ± 0.09	1.69 ± 0.04	1.72 ± 0.07	1.54 ± 0.11
Peak-peak (µV)	84.1 ± 8.5	115.4 ± 21.4	113.6 ± 6.6	170.6 ± 18.4	179.7 ± 30.3
durPA (ms)	1.98 ± 0.08	2.03 ± 0.09	2.16 ± 0.04	2.25 ± 0.06	2.11 ± 0.12
dVmax (mV/s)	5.4 ± 0.6	8.2 ± 1.3	7.7 ± 0.4	11.4 ± 1.2	11.5 ± 1.7
dVmin (mV/s)	−4.8 ± 0.5	−6.3 ± 0.8	−5.6 ± 0.3	−7.9 ± 0.9	−9.2 ± 1.6

**Table A4:** Properties of mAP for P1N1P2. Values as $\bar{x}\pm SEM$, except of *N*, which indicates the number of mAP picked up from every nucleus.

Properties	Ce.pc	Ce.mc	V.c	V.im	DD.NN
N	1	5	5	4	2
N1 (µV)	-	8.8 ± 0.6	7.2 ± 2.2	5.3 ± 3.2	-
P1 (µV)	-	−106.1 ± 6.8	−88.8 ± 22.6	−123.8 ± 36.4	-
N2 (µV)	-	32.1 ± 3.2	50.2 ± 15.8	62.3 ± 13.8	-
durN1 (ms)	-	0.10 ± 0.00	0.14 ± 0.03	0.01 ± 0.04	-
durP1 (ms)	-	0.27 ± 0.00	0.57 ± 0.12	0.48 ± 0.10	-
durN2 (ms)	-	2.11 ± 0.09	1.85 ± 0.19	1.98 ± 0.07	-
Peak–peak (µV)	-	138.2 ± 9.9	138.9 ± 37.8	186.1 ± 50.0	-
durPA (ms)	-	2.39 ± 0.09	2.56 ± 0.15	2.47 ± 0.12	-
dVmax (mV/s)	-	10.3 ± 0.7	8.4 ± 2.0	7.6 ± 0.9	-
dVmin (mV/s)	-	−11.0 ± 0.7	−7.4 ± 1.6	−8.7 ± 1.6	-

durN1 = duration of depolarization phase; durP1 = duration of repolarization phase; N1 = depolarizing amplitude, P1 = repolarizing amplitude; Peak-peak = peak-to-peak amplitude; durPA = duration of whole of the AP; dVmax = maximum value of first derivative; dVmin = minimum value of first derivative.

**Table A5:** Values for the first three phases of repolarization. Values as $\bar{x}\pm SEM$, except of *N*, which indicates the number of mDAP.

		Phases During Repolarization (mV/s)		
Properties	*N*	*m*_1_	*m*_2_	*m*_3_
P1P2N1	768	21.10 ± 0.72	13.08 ± 0.48	18.08 ± 0.90
P1N2	69	18.54 ± 2.36	7.26 ± 1.99	10.08 ± 2.55
N1P1N2	212	24.78 ± 1.44	11.03 ± 0.91	14.33 ± 1.31
P1N1P2	15	−43.84 ± 6.21	−9.66 ± 2.14	−17.75 ± 7.70
